# Supplementary material for: Learning curve and surgical outcome of robotic assisted colorectal surgery with ERAS program
Source: Sci Rep. 2022 Nov 29;12:20566. doi: 10.1038/s41598-022-24665-w (PMC9709162; doi:10.1038/s41598-022-24665-w)
Supplement: Supplementary file 2 — Supplementary Information 2. [file 41598_2022_24665_MOESM2_ESM.zip › RAL Raw data-Ñ[▒K/104/OP N_ñw╝╨░O▒Kñσ.pdf]

W95 / W95

臺中榮民總醫院
手術室護理記錄

[redacted]

基本資料

- 室別： 開刀房-17室，手術科別： CRS，手術類別： 常規，日期： 2021/08/12
- 進入時間： 0730 ； 麻醉開始時間： 0735 ； Time Out時間： 0905 ； 劃刀時間： 0906 ； 手術結束時間： 1635 送出時間： 1650 ；
- 麻醉方式： GE

  診斷： CA OF RECTUM

  手術名稱： 1.Robot Assisted Low Anterior Resection
  2.Debridement for wound(deep) 5-10cm

  手術局部用藥： nil

  術前預防性抗生素：
  - 抗生素名稱一： Veterin 1GM IVD由麻醉護理師給予
  - 時間： 0855
  - 上次簽章: 鄭鈞躍

  ，追加預防性抗生素：
  - 抗生素名稱一： Veterin 1GM IVD由麻醉護理師給予
  - 時間： 1255
  - 上次簽章: 鄭鈞躍
- 手術醫師： 蔣鋒帆，呂俊德，楊曙榮，陳璿夫，洪浚嚴，陳奕嘉，陳咨言
- 麻醉醫師： VS 張思玲
-
  - 刷手護士A ： 歐真勤，時間： 0730，刷手護士B ： 許芷瑄，時間： 0730，接替刷手一： 鄭鈞躍，時間： 1130，接替刷手二： 蘇敬涵，時間： 1200
  - 流動護士A ： 鄭鈞躍，時間： 0730，接替流動一： 許芷瑄，時間： 1100，接替流動二： 鄭鈞躍，時間： 1200
- 更多接替及流動護士： 接替刷手三： 陳盈妤，時間： 1500，接替流動三： 蘇敬涵，時間： 1500

皮膚狀況

A-V Shunt： 無

術前： 不完整

部位(術前)：

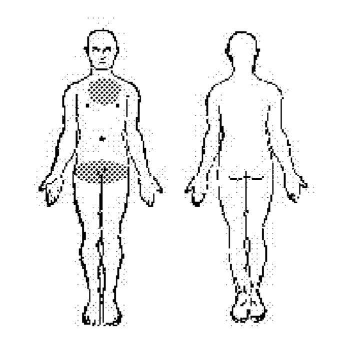

胸前紅疹、雙側腹股溝傷口

記錄者： 上次簽章: 鄭鈞躍

術後： 不完整

部位(術後)：

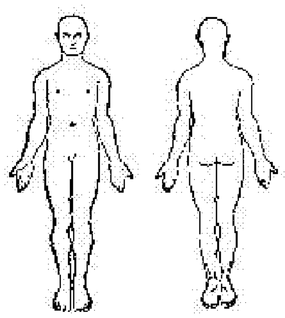

同術前

記錄者：

手術準備

手術臥式

-
  - 手術臥式一： 截石臥式
  - 使用附件： 腳架，其它： CRS Lateral support
  - 皮膚準備： 10%Aq Betadine，Hibiscrub，其它： 2% chlorhexidine
  - 電燒： 使用電燒: 電極片位置： 其它： 迴路電極墊，記錄者： 蘇敬涵
-
  - 手術臥式二： 仰臥
  - 使用附件：
  - 皮膚準備： 10%Aq Betadine
  - 電燒： 使用電燒: 電極片位置： 其它： 迴路電極墊，記錄者： 蘇敬涵

止血帶T： 無

植入物

- 植入物名稱/號碼/數量/部位： davinci Endo GIA/45-4.3 x3/colon
  ethicon EEA 33mm/colon
  Covidien GIA/80-3.8 x1/colon

, 記錄者： 上次簽章: 鄭鈞羅

標本送檢

- 細菌培養 一般，採取部位: 1. 右腹股溝傷口，數量： 1
- 細胞學 無
- 冰凍切片 無
- 病理標本
  組織名稱一：rectal
  ,
  組織名稱二：IMA root
  ,
  組織名稱三：distal cut end
  , 更多組織名稱
  - 組織名稱四：parearotic LN
  - 組織名稱五：peritoneum
- 截肢 無

標本交付

-
  - 標本： 5 袋
  - 標本： 0 盒
  - 標本： 0 瓶
  - 細胞學： 0 管
  - Culture Tube： 0 管
  - 被交付醫師： R 洪浚嚴
  -
    - 記錄者： 上次簽章: 鄭鈞羅
    - 時間： 1410

-
  - 標本： 0 袋
  - 標本： 0 盒
  - 標本： 0 瓶
  - 細胞學： 0 管
  - Culture Tube： 1 管
  - 被交付醫師： CR 陳奕嘉
  -
    - 記錄者： 上次簽章: 蘇敬涵
    - 時間： 1610

傷口術後敷料

傷口敷料：　　　　　　　　　　　　　傷口敷料： 無

- 請於圖上標示部位：

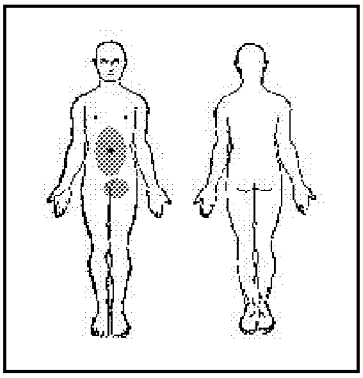

- 傷口敷料： 腹部:Trocar洞 menpore
  腹股溝:紗布

導管

導管	天數	導管資訊	插入日期	插入時間	更換日期	拔除日期	拔除時間	拔除原因	預計拔除/更換	預計拔除/更換日期
Foley(1)	1	號碼:16 Fr.; 部位:膀胱;	2021/08/12	0810						
A-line (1)	1	號碼:20 G; 部位:左橈動脈;	2021/08/12	0815						
Penrose Drain(1)	1	號碼:12 mm.; 部位:骨盆腔;	2021/08/12	1345						
Double Lumen CVC(1)	1	號碼:8 Fr; 深度:15cm; 部位:jugular (右); 象限:RU;	2021/08/12	0820						
Epidural PCA(1)	1	部位:硬脊膜;	2021/08/12	0800						
Chemo Port(1)	172	號碼:8.5 Fr.; 部位:左胸;	2021/02/22	2200						

用物計數

- 手術中出血量： 100 c.c.
- 手術中尿量： 800 c.c.
-
  - 其它： 無

用物計數：

- 部位一： 腹部，1.器械： 正確，2.縫針： 正確，3.刀片： 正確，4.紗布： 正確，5.腹部墊： 未使用，6.紗球： 未使用，7.PEANUTS： 未使用，8.紗條： 未使用，9.絲帶： 未使用，10.針頭： 未使用，11.Vessel loop： 未使用，12.Boots： 未使用，13.Jelco： 未使用，14.棉條： 未使用，
  - 第一次：
    - 刷手護士 ： 蘇敬涵
    - 流動護士： 上次簽章: 鄭鈞躍
    - 時間： 1355

,
  - 第二次：

- 刷手護士 ： 蘇敬涵
- 流動護士： 上次簽章: 鄭鈞躍
- 時間： 1410

,

- 第三次：
  - 刷手護士 ： 蘇敬涵
  - 流動護士： 上次簽章: 鄭鈞躍
  - 時間： 1415

- 部位二： 腹股溝，1.器械： 正確，2.縫針： 正確，3.刀片： 正確，4.紗布： 正確，5.腹部墊： 未使用，6.紗球： 未使用，7.PEANUTS： 未使用，8.紗條： 未使用，9.絲帶： 未使用，10.針頭： 未使用，11.Vessel loop： 未使用，12.Boots： 未使用，13.Jelco： 未使用，14.棉條： 未使用，
  - 第一次：
    - 刷手護士 ： 陳盈妤
    - 流動護士： 上次簽章: 蘇敬涵
    - 時間： 1606

  ,
  - 第二次：
    - 刷手護士 ： 陳盈妤
    - 流動護士： 上次簽章: 蘇敬涵
    - 時間： 1615

  ,
  - 第三次：
    - 刷手護士 ： 陳盈妤
    - 流動護士： 上次簽章: 蘇敬涵
    - 時間： 1625

-
  - 第一次：
    - 流動護士：

  ,
  - 第二次：
    - 流動護士：

  ,
  - 第三次：
    - 流動護士：

備註

備註： Davinci 30度 scope No.2

- 記錄者： 上次簽章: 鄭鈞躍
- 時間： 0730

填寫護理人員 蘇敬涵

正式摘要

W95 / W95

doctorRank:B
